# Recent Mechanistic Studies in Allergic Diseases

**DOI:** 10.3390/ijms241814312

**Published:** 2023-09-20

**Authors:** Sayantani B. Sindher, Reyna Sharma, Medha Yarlagadda, Andrew R. Chin, R. Sharon Chinthrajah

**Affiliations:** Department of Medicine, Sean N. Parker Center for Allergy and Asthma Research at Stanford University, Stanford, CA 94305, USA; tina.sindher@stanford.edu (S.B.S.); reynas12@stanford.edu (R.S.); 24medhay@students.harker.org (M.Y.); andychin@stanford.edu (A.R.C.)

Allergic diseases, such as food allergies, asthma, and allergic rhinitis, continue to present a significant challenge for a broad cross-section of the population, despite recent advancements in their treatment and prevention. This underscores the crucial need for a deeper understanding of the mechanisms stimulating allergic diseases. The recent Special Issue in *IJMS*, entitled “Immunometabolism and Immunologic Mechanisms in Allergy Diseases”, features two excellent reviews that delve into the mechanisms implicated in cow’s milk allergies and mast cell responses in allergic diseases. Additionally, the Special Issue includes four research papers that explore recent advances in understanding the molecular mechanisms promoting allergic diseases, elucidating novel therapeutic strategies that could be employed for the better management and treatment of allergic disease.

Emmert et al. comprehensively review the immune mechanisms driving cow’s milk allergy, one of the most prevalent food allergens, in their review article entitled “Current Practice in Pediatric Cow’s Milk Protein Allergy: Immunological Features and Beyond” [1]. They discuss how milk protein allergy (CPMA) is categorized into two subtypes: IgE and non-IgE mediated allergies. IgE-mediated CPMA symptoms take effect almost immediately after the consumption of milk, most commonly affecting the skin or the gastrointestinal and respiratory tracts. On the other hand, non-IgE-mediated CPMA symptoms are more delayed, with onset occurring anywhere between hours and weeks. The symptoms fall into three main clinical conditions, namely food-protein-induced enterocolitis syndrome (FPIES), food-protein-induced enteropathy (FPE), and food-protein-induced allergic proctocolitis (FPIAP). The two main protein fractions of milk, coagulum containing casein proteins and lactose rum, or whey proteins, contain compounds that result in a greater allergenic prevalence. On a molecular level, IgE-mediated CPMA reflects the degranulation of innate immune cells or the immediate release of histamine, especially after the repeated exposure of food-specific IgE. This IgE, in turn, attaches to Fc-Epsilon-Receptor 1 (FcεR1) on mast cell and basophil cell surfaces, resulting in an allergic reaction. In terms of diagnosis, the optimum methods currently employed include the skin prick test, the measurement of specific serum IgE levels, component-resolved allergy testing, and the basophil activation test. Current treatment methods include a range of immunotherapy procedures. Emmert et al. emphasize the importance of ongoing research into both IgE and non-IgE-mediated CPMA and its impact on allergic individuals. Enhancing preventative strategies and treatments remains essential to reducing the escalating strain that CPMA places on the healthcare sector and affected families.

Mast cells play a critical role as effector cells in allergic diseases, such as food allergy and asthma. Haque and Frischmeyer-Guerrerio provide a comprehensive review of the multifaceted role of mast cells in various biological processes within allergic diseases. These processes include protection against venom poisoning, infections, autoimmune conditions, wound healing, angiogenesis, and fibrosis, as discussed in their review article entitled “The Role of TGFβ and Other Cytokines in Regulating Mast Cell Functions in Allergic Inflammation” [2]. In their review, the authors underscore the significance of cytokines in regulating mast cell effector functions and allergic inflammation. Many of these cytokines have been identified as potential therapeutic targets. The authors review the complex and still not fully understood role of TGFβ signaling in mast cell function. This includes its role in inducing mast-cell-mediated regulatory T cell function, the regulation of mast cell development, survival, and effector activity. The authors also discuss the involvement of other cytokines, including IL-10, IL-35, IL-37, and IL-33, which have been identified as potential regulators of mast cell function. They briefly discuss the role of IL-10 in mediating mast cell effector responses in allergic diseases. While both IL-35 and IL-37 are known to suppress mast cells, they do so through different mechanisms and have been studied in differing disease contexts. Specifically, IL-35 has been studied in the context of allergic rhinitis and asthma, while IL-37 has been demonstrated to primarily interact with mast cells in the skin. Finally, the authors discuss the complex role of IL-33 in regulating mast cell function, which tends to potentiate mast cell responses. Overall, this review highlights that there is an intricate interplay between cytokines, especially TGFβ, and mast cell functions in allergic reactions. It emphasizes the potential application of cytokines as therapeutic targets for managing allergic diseases and underscores the need for further research in order to unravel their precise mechanisms and roles. An enhanced understanding of the pathways that control mast cell effector activity in allergic diseases could lead to the development of novel therapeutic strategies.

In addition to these reviews discussing the mechanisms implicated in allergic diseases, this Special Issue also includes four research articles that provide deeper insights into the mechanisms underlying allergic responses to various exposures and responses. In their research article entitled “Effects of Oral Exposure to Low-Dose Bisphenol S on Allergic Asthma in Mice” [3], Yanagisawa et al. investigate the impact of oral exposure to low doses of Bisphenol S (BPS) (0.04, 0.4, and 4 μg/kg/day) on allergic asthma in mice. Bisphenols, including the commonly used Bisphenol A (BPA), are found in polycarbonate plastic and epoxy resins. Due to the adverse effects of BPA on human health, including the development and exacerbation of allergic responses in asthma, BPA has been banned by several health organizations. This has led to the increasing application of BPS as an alternative. However, the potential impact of BPS on diseases such as allergic asthma has not been well studied. Therefore, Yanagisawa et al. use an ovalbumin (OVA)-sensitized allergic asthma mouse model to investigate the potential impact of BPS in allergic asthma. They found that concurrent exposure to BPS with OVA exacerbated allergic pulmonary inflammation, Th2 cytokine and chemokine production, and serum OVA-specific IgE secretion when compared to exposure to OVA alone. Interestingly, this effect was more pronounced in the moderate (0.4 µg/kg/day) group than in the heavy (4 µg/kg/day) exposure group. BPS exposure led to an elevation in the protein expression levels of Th2 cytokines (IL-5, IL-13), IL-33 and CCL11/Eotaxin in the lungs of OVA-treated mice. In contrast, Th1 cytokines like IFN-y showed no significant changes. This study suggests that BPS may enhance allergic asthma symptoms by activating type 2 immunity. For example, IL-5 promotes eosinophil growth, differentiation, activation, and survival, while IL-13 induces B-cell IgE class switching and music hypersecretion. IL-33, an innate immune activator, contributes to both innate and adaptive immunity. CCL11/Eotaxin is associated with eosinophilic infiltration in various inflammatory diseases. Furthermore, this article also highlights the potential involvement of Estrogen Receptors (ERs). BPS, a known endocrine-disrupting chemical (EDC), may exert its effects by altering ERs, which regulate the immune and endocrine systems. The research suggests that BPS might exacerbate allergic asthma by affecting ER activation in the lungs, with varying effects depending on the exposure dose. Additionally, BPS exposure could suppress anti-inflammatory responses as it reduces G Protein-Coupled Estrogen Receptor (GPER) expression in the lungs, which potentially contribute to allergic asthma exacerbation. BPS exposure has been found to increase the activation of antigen-presenting cells (APCs) in the mediastinal lymph nodes (MLNs) of OVA-sensitized mice. It activates dendritic cells (cDC2 subset) that induce Th2 immune responses, potentially promoting Th2 cell migration from MLNs to the lungs and worsening allergic asthma. This study demonstrates that of the exposure of humans to BPS could exacerbate allergic asthma via multiple mechanisms, including the induction of Th2 cytokines, immune cell activation, and the interaction with estrogen receptors. This underscores the need to understand the risks of BPA alternatives, including BPS, in maintaining human health.

In addition to the components of plastics and resins, maternal asthma and exposure to environmental factors like air pollution and cigarette smoke are strongly associated with the risk of asthma in children. This suggests that maternal exposure can influence the developing immune system of the child via epigenetic mechanisms, possibly triggered by the maternal immune system. In asthma, the production of specialized pro-resolving mediators (SPMs), which control the mediators responsible for the resolution of inflammation, is impaired, leading to prolonged allergic inflammation in asthma. In this Special Issue, Ramar et al. discuss how the application of the synthetic SPMs Lipoxin A4 (LxA4) and Resolvin E2 (RvE2) can protect against maternal exposure to air pollutants; this is explored in their research article entitled “Intra-Airway Treatment with Synthetic Lipoxin A4 and Resolvin E2 Mitigates Neonatal Asthma Triggered by Maternal Exposure to Environmental Particles” [4]. Maternal exposure to urban air particles (CAPs) and diesel exhaust particles (DEPs) results in a heightened susceptibility to allergens in the offspring, leading to an eosinophilic response even when the offspring are not directly exposed to the inflammatory stimulus. Intra-airway treatment with synthetic LxA4 or RvE2 has been found to reduce eosinophilia, lung tissue infiltration, and pro-allergic cytokine levels in offspring born to mothers exposed to CAPs and DEPs. These findings suggest that SPM therapies might mitigate the impact of hazardous environmental exposures, like particle pollution, that contribute to asthma development. These therapies have the potential to target asthma triggered by environmental factors, without affecting normal immune responses. The therapeutic effects of the SPMs evaluated in this study complement prior findings in allergen-induced asthma models and highlight the potential of these mediators to act as immune-resolving therapies.

Another exciting anti-allergic agent is fucoidan, which is a fucose-rich sulfated polysaccharide extracted from brown sea algae that effectively reduces type 1 allergic hypersensitivity. Previous studies have indicated that the oral administration of Fucoidan releases Gal9 into the blood, removing IgE bound to mast cells and therefore inhibiting allergy development and mast cell activation. However, 12 g of seaweed would need to be consumed daily for fucoidan to significantly reduce the development of allergies, which is difficult to achieve. Therefore, in an article entitled “Anti-Allergic Activity of Fucoidan Can Be Enhanced by Coexistence with Quercetin” [5], Mizuno et al. delve into introducing other food factors in order to enhance the anti-allergenic functionality of fucoidan, so that it can be consumed in smaller amounts while its positive effects continue to be implemented. The authors find that the consumption of foods containing quercetin and kaempferol induces an anti-allergic response, enabling a reduction in the amount of fucoidan needed to almost half of that initially deemed necessary in an allergic mouse model. This study highlights the potential benefits of combining multiple therapeutics, which could enable good outcomes to be achieved with lower doses of therapeutics.

The activation of bitter taste receptors releases nitrogen oxide, which maintains homeostasis in airways; this is thought to be dysregulated in allergic diseases such as chronic rhinosinusitis. However, the mechanisms by which these receptors contribute to disease are not well understood. In their article, entitled “Functional Alteration and Differential Expression of the Bitter Taste Receptor T2R38 in Human Paranasal Sinus in Patients with Chronic Rhinosinusitis” [6], Takemoto et al. study the role of two bitter taste receptors known to induce immune response, T2R14 and T2R38, in chronic rhinosinusitis. T2R38 is essential for sinonasal epithelial defense against respiratory bacterial infection, suggesting its potential role in chronic rhinosinusitis. The authors evaluate the expression of T2R14 and T2R38 in both eosinophilic and non-eosinophilic chronic rhinosinusitis. T2R38 was found to be significantly downregulated in the ethmoid mucosa of non-eosinophilic chronic rhinosinusitis patients and the nasal polyps of eosinophilic chronic rhinosinusitis patients, suggesting variations in the role of bitter taste receptors in different disease endotypes. Within these tissues, T2R38 expression was found primarily in epithelial ciliated cells, with little to no staining in secretary goblet cells. These findings suggest the previously uncharacterized role of bitter taste receptors in chronic rhinosinusitis and pave the way for future studies, aiming to determine how these mechanisms influence allergic diseases.

In summary, this Special Issue encompasses two comprehensive reviews examining the broader mechanism underlying allergic diseases, as well as four research articles providing in-depth insights into the molecular mechanisms promoting specific allergic triggers and responses. These articles identify key areas of therapeutic focus that could lead to innovations in therapeutic strategies.
